# Changes in the Airborne Bacterial Community in Outdoor Environments following Asian Dust Events

**DOI:** 10.1264/jsme2.ME13080

**Published:** 2014-02-19

**Authors:** Nobuyasu Yamaguchi, Jonguk Park, Makiko Kodama, Tomoaki Ichijo, Takashi Baba, Masao Nasu

**Affiliations:** 1 Graduate School of Pharmaceutical Sciences, Osaka University, 1–6 Yamadaoka, Suita, Osaka 565–0871, Japan

**Keywords:** bioaerosol, airborne bacteria, Asian dust, long-range transportation, bacterial community analysis

## Abstract

Bacterial abundance and community compositions have been examined in aeolian dust in order to clarify their possible impacts on public health and ecosystems. The influence of transcontinentally transported bacterial cells on microbial communities in the outdoor environments of downwind areas should be determined because the rapid influx of a large amount of bacterial cells can disturb indigenous microbial ecosystems. In the present study, we analyzed bacteria in air samples (approximately 100 m^3^ d^−1^) that were collected on both Asian dust days and non-Asian dust days over 2 years (between November 2010 and July 2012). Changes in bacterial abundance and community composition were investigated based on their 16S rRNA gene amount and sequence diversity. Seasonal monitoring revealed that airborne bacterial abundance was more than 10-fold higher on severe dust days, while moderate dust events did not affect airborne bacterial abundance. A comparison of bacterial community compositions revealed that bacteria in Asian dust did not immediately disturb the airborne microbial community in areas 3,000–5,000 km downwind of dust source regions, even when a large amount of bacterial cells were transported by the atmospheric event. However, microbes in aeolian dust may have a greater impact on indigenous microbial communities in downwind areas near the dust source. Continuous temporal and spatial analyses from dust source regions to downwind regions (*e.g.*, from the Gobi desert to China, Korea, Japan, and North America) will assist in estimating the impact of atmospherically transported bacteria on indigenous microbial ecosystems in downwind areas.

Bacterial migration is a natural phenomenon promoted by ocean currents and atmospheric events. Aeolian dust is primarily found in arid and semi-arid regions. When a wind-sand stream occurs, microorganisms on dust particles can be lifted and transported over long distances by air currents. Major aeolian dust events arise from the Sahara and Sahel deserts (African dust), Australian deserts (Australian dust), and the Taklamakan Desert, Gobi Desert, and Loess Plateau (Asian dust). Bacterial abundance and community compositions have been examined in these aeolian dusts to clarify their possible impacts on public health and ecosystems (6–8, 10, 18).

Approximately 4,000,000 tons of Asian dust particles have been estimated to fall on Japan annually (24), which is 3,000–5,000 km from their source regions (China and Mongolia). Asian desert dust particles are known to be transported long distances (13, 14), and even reach the North American Continent (more than 15,000 km away) (4, 16). Moreover, oceanic deposition has been shown to enhance phytoplankton growth in the North Pacific Ocean via natural iron fertilization (1). Previous studies reported that Asian dust particles can sometimes be transported globally in 13 days (21) and have been identified in the ice and snow cores of Greenland (2) and the French Alps (9). We collected Asian dust particles using a small airplane, and subsequent bio-imaging and 16S rRNA gene analysis revealed that these samples carried phylogenetically diverse bacterial cells for thousands of kilometers, some of which retained growth potential in spite of the long distances they were transported (23). Accordingly, the influence of these transcontinentally transported bacterial cells on microbial communities in outdoor environments of downwind areas should be determined because the rapid influx of a large amount of bacterial cells has the potential to disturb indigenous microbial ecosystems.

In the present study, we collected approximately 100 m^3^/d of air over 2 years (between November 2010 and July 2012) on both Asian dust days and non-Asian dust days (background) and analyzed the samples for changes in the abundance and community composition of airborne bacteria. Airborne bacterial concentrations were measured by quantitative PCR targeting the eubacterial 16S rRNA gene, and both culturable and non-culturable airborne bacterial species were estimated based on their 16S rRNA gene sequences.

## Materials and Methods

### Sample collection

Aerosol samples were collected on the rooftop of a building (*ca.* 20 m in height) of Osaka University in Osaka, Japan (latitude: N34°9′1.89″, longitude: E135°31′15.61″) using an originally fabricated dust sampler that consisted of wet glass beads in a stainless steel can (volume: 2 L) and a sterilized Teflon inlet tube (23). A high-volume vacuum pump pulled 120 L min^−1^ of air into the sampler, and aerosol particles in the air were adsorbed onto the surface of the wet glass beads in the sampler. Aerosol particles from a total of approximately 100 m^3^ of ambient air were collected during each sampling event (sampling time: approximately 14 h). Fifteen samples were obtained on different days between November 2010 and July 2012, including severe Asian dust days (12 November 2010 and 2 May 2011) and moderate Asian dust days (10 April 2011, 24 April 2012, and 16 May 2012). The occurrence of atmospheric Asian dust was confirmed and the severity of the Asian dust event was also defined by information from the Japan Meteorological Agency, LIDAR (Light Detection and Ranging) data from the Ministry of the Environment, Japan (http://www-gis5.nies.go.jp/eastasia/DustLider.php), and visibility at the sampling location (Supplementary Fig. S1). Aerosol samples collected on wet beads were suspended in 2.5 L of particle-free water and used for the following experiments.

### Direct DNA extraction

Aerosol samples (2 L of the 2.5 L suspension) were filtered onto 0.4-μm pore size sterilized polycarbonate membrane filters (Toyoroshi, Tokyo, Japan). DNA was then extracted and purified using the method described by Tsai and Olson (20).

### Cultivation of bacteria in aerosol samples and DNA extraction

Aerosol samples (1 mL of the 2.5 L suspension) were inoculated into 9 mL of PYG broth (5 mg mL^−1^ peptone, 2.5 mg mL^−1^ yeast extract, and 1 mg mL^−1^ glucose), 1% PYG broth, 0.05% yeast extract, or R2A broth (Nihon Pharmaceutical, Osaka, Japan). Samples were then incubated at 25°C for 7 d with slow shaking, after which bacterial DNA was extracted and purified using the Wizard Genomic DNA Purification Kit (Promega, Madison, WI, USA) according to the manufacturer’s instructions. The final sample elution volume was 25 μL in TE buffer (10 mM Tris-HCl and 1 mM EDTA [pH 8.0]).

### Amplification of 16S rRNA using nested-PCR for DGGE analysis

Nested-PCR was performed to amplify the 16S rRNA gene for DGGE analysis. In the first round of PCR, nearly full-length 16S rRNA gene sequences of the bacterial domain were amplified using the universal primers 8f (5) and 1492r (22). PCR amplification was performed using the reagents supplied with AmpliTaq Gold (Applied Biosystems, Carlsbad, CA, USA). The reaction mixture contained 0.05 U mL^−1^ AmpliTaq Gold, 0.5 μM of each primer, 0.2 mM of each dNTP, 1.5 mM MgCl_2_, 25 μg mL^−1^ 8-methoxypsoralen (dissolved in dimethyl sulfoxide; Sigma Aldrich, St. Louis, MO, USA), and 0.1 mg mL^−1^ bovine serum albumin (Takara Bio, Shiga, Japan) in the PCR buffer (49 μL). A 1 μL DNA suspension was finally added after irradiation of the PCR mixture with UV light (15). The reaction cycle consisted of an initial denaturing step at 95°C for 15 min, followed by 25 cycles of denaturing at 95°C for 1 min, annealing at 42°C for 1 min, and extension at 72°C for 2 min, with a final extension step at 72°C for 10 min. Primary PCR products were then purified using the MonoFas PCR Purification Kit (GL Sciences, Tokyo, Japan) and eluted with 40 μL of TE buffer. The variable V6–V8 regions of the 16S rRNA gene fragments were then amplified by a second round of PCR using the primers GC-clamp-EUB f933 and EUB r1387 (11). The PCR mixture contained 0.05 U/ml AmpliTaq Gold, 0.4 μM of each primer, 0.2 mM of each dNTP, 3 mM MgCl_2_, 12.5 μg mL^−1^ 8-methoxypsoralen, and 0.2 mg mL^−1^ BSA in the PCR buffer (98 μL). A 2 μL DNA suspension was finally added after irradiation of the PCR mixture with UV light (15). The reaction cycle consisted of an initial denaturing step at 95°C for 10 min, followed by 20 cycles of denaturing at 95°C for 1 min, annealing for 1 min at 66°C to 56°C (decreased by 0.5°C per cycle), and extension at 72°C for 3 m. Five additional cycles were performed as follows: denaturing at 95°C for 1 min, annealing at 56°C for 1 min, and extension at 72°C for 3 min with a final extension step at 72°C for 7 min.

### DGGE analysis

Secondary PCR products were purified and concentrated using the MonoFas PCR Purification Kit (GL Sciences), followed by elution with 20 μL of DNA-free water. DNA concentrations were determined using a Biospec-Nano Spectrophotometer (Shimadzu, Kyoto, Japan). Briefly, 150–200 ng of DNA was loaded onto a 6.5% (w/v) polyacrylamide gel (acrylamide/bisacrylamide [37.5:1]) in 1× TAE buffer (40 mM Tris-HCl, 20 mM acetic acid, 1 mM EDTA [pH 8.0]) with a denaturing gradient ranging from 40 to 70%. The denaturant (100%) consisted of 7 M urea and 40% formamide. Electrophoresis was conducted at 55°C for 10 min at 20 V, followed by 16 h at 80 V. After electrophoresis, the gels were stained with 1× SYBR Gold nucleic acid gel stain (Invitrogen, Carlsbad, CA, USA), and digital images were captured using the Gel Doc XR system (Bio-Rad Laboratories, Hercules, CA, USA).

### Sequencing of DGGE fragments

All bands in the DGGE gel of 16S rRNA fragments were excised with a razor blade and soaked in DNA-free water at 4°C overnight. The DNA sample was then subjected to PCR amplification using the EUB f933 and EUB r1387 primers (11). PCR products were then purified with the MonoFas PCR Purification Kit and cloned into the pGEM-T-easy Vector System (Promega) using *Escherichia coli* JM109 as the host. The clones were then subjected to PCR using the M13 primers. The diluted PCR products were then further amplified using the GC-clamp-EUB f933 and EUB r1387 primers, after which DGGE was performed. Clones were sequenced and then phylogenetically analyzed. The sequencing of selected clones was performed on a CEQ8000 DNA Genetic Analysis System (Beckman Coulter, Indianapolis, IN, USA) with M13 primers. Base-calling of sequence trace data was performed using the CodonCode Aligner version 3.7.1 (CodonCode Corporation, Dedham, MA, USA). Sequences were aligned using Clustal W, and phylogenetic trees were constructed using the Maximum-likelihood method. Sequence homologies were searched with the blastn program from the DNA Data Bank of Japan (DDBJ; http://www.ddbj.nig.ac.jp).

### Random cloning

Genomic libraries were constructed from the following four samples: 12 November 2010 and 2 May 2011 (severe Asian dust days), and 18 January 2012 and 17 July 2012 (non-Asian dust days). To accomplish this, PCR products amplified using the universal primers EUBf933 and EUBr1387 were cloned into the pGEM-T-easy Vector System. The 16S rRNA gene fragments of a hundred clones were then randomly selected from each aerosol sample and sequenced by Fasmac (Greiner Japan, Tokyo, Japan). These sequences were analyzed by the ribosomal database project (3).

### Quantitative real-time PCR

To determine bacterial abundance, 16S rRNA gene occurrence was quantified by real-time PCR using a LightCycler (Roche Diagnostics, Mannheim, Germany). Real-time PCR was performed with eubacterial primer sets according to the procedure described by Yamaguchi *et al.* (23). A total of 1 × 10^1^ to 1 × 10^7^ copies/reaction of PCR products of *E. coli* W3110 were used as the standard DNA template to generate a standard curve to quantify the 16S rRNA gene.

### Nucleotide sequence accession numbers

The sequences obtained in this study were deposited in the DNA Data Bank of Japan (DDBJ) and assigned accession numbers AB776048 to AB776648.

## Results and Discussion

### Changes in bacterial abundance in outdoor environments following Asian dust events

The abundance of airborne bacteria was determined by quantitative PCR targeting the eubacterial 16S rRNA gene (Table 1) in samples collected during severe dust events (A1 and A3), moderate dust events (A2, A4, and A5), and on non-Asian dust days (N1 to N10). The source of dust particles during these severe Asian dust events was estimated to be the Gobi desert (Supplementary Fig. S2). Seasonal monitoring of airborne bacterial abundance revealed that it was more than 10-fold higher on severe dust days, while moderate dust events did not affect airborne bacterial abundance. Bacterial abundance on non-Asian dust days was less than 10^4^ cells m^−3^ and seasonal variations in airborne bacterial abundance were not observed.

### Changes in the airborne bacterial community in outdoor environments following Asian dust events

**Fig. 1:**
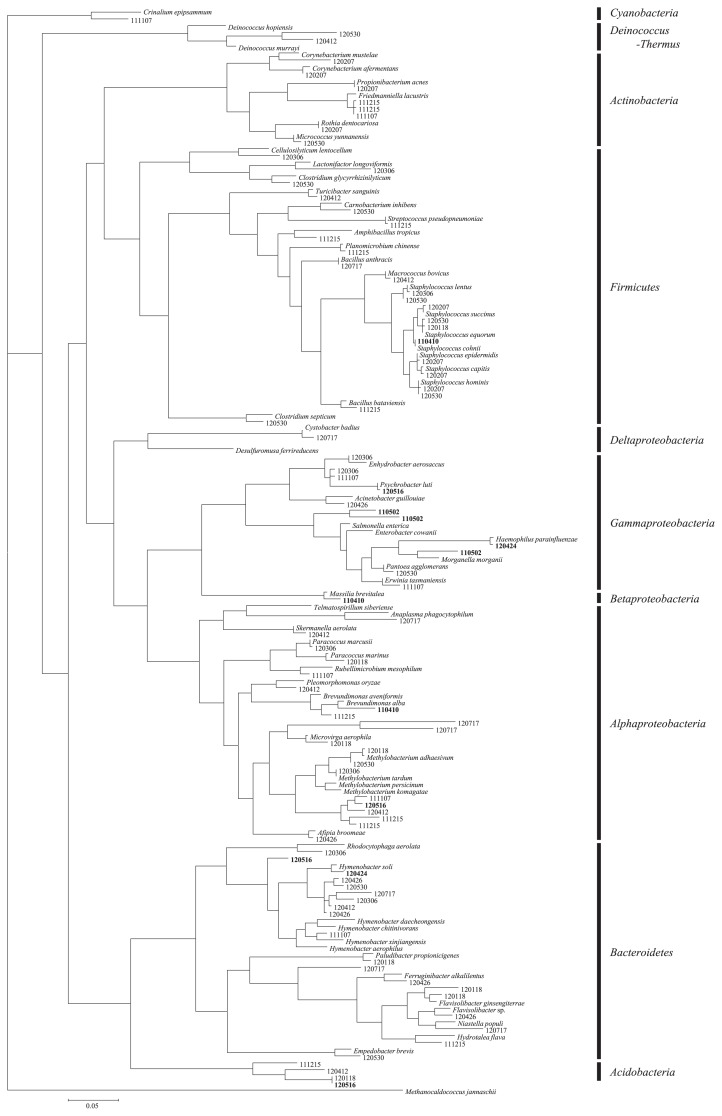
Maximum-likelihood phylogenetic tree of airborne bacteria based on the 16S rRNA sequences of DGGE fragments. Bacteria collected on Asian dust days are shown as bold characters.

**Fig. 2:**
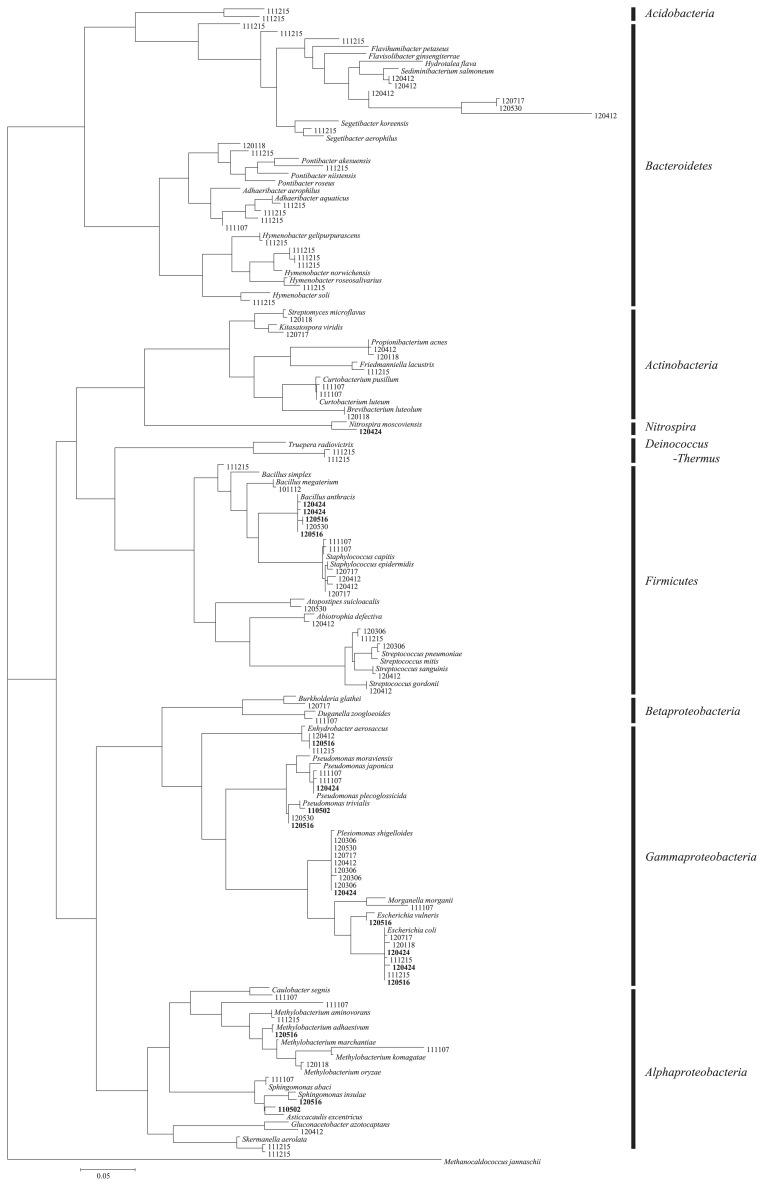
Maximum-likelihood phylogenetic tree of culturable airborne bacteria based on the 16S rRNA sequences of DGGE fragments. Bacteria collected on Asian dust days are shown as bold characters.

DGGE was used to estimate changes in the airborne bacterial community following Asian dust events. The sequences of 189 bands on DGGE gels were determined (14 bands from uncultured bioaerosol samples collected on Asian dust days, 18 bands from cultured samples collected on Asian dust days, 78 bands from uncultured samples collected on non-Asian dust days, and 79 bands from cultured samples collected on non-Asian dust days). Negative control samples (particle-free sterilized water) were analyzed and the results obtained confirmed that no PCR products were detected in any negative control sample after nested PCR. Phylogenetically diverse bacteria were confirmed to exist in the outdoor air and airborne bacteria found during Asian dust events (*Firmicutes*, *Bacteroidetes*, *Actinobacteria*, and *Proteobacteria*) belonged to the same phyla present on non-Asian dust days (background air) (Fig. 1). We focused on culturable airborne bacteria to investigate what species maintained growth potential following atmospheric transport (Fig. 2). Although no specific bacteria were found to be associated with Asian dust events, *Firmicutes* and *Proteobacteria* were more frequently detected in cultured bioaerosol samples (Fig. 2) than in uncultured bioaerosol samples (Fig. 1) collected on Asian dust days. *Firmicutes* (*Bacillus* spp.) found in Asian dust is generally found in dust source regions (23) and can form spores to facilitate atmospheric survival. Some *Proteobacteria* found in Asian dust were similar to *Sphingomonas* spp., which is found in sea water. Asian dust often carries a mixture of dust particles and marine aerosols to Japan (12) because the dry stream of Asian dust absorbs wet marine aerosols when it passes over the Sea of Japan. Therefore, *Proteobacteria* may be recent dust contaminants via marine aerosols and, as such, can maintain their viability.

**Fig. 3:**
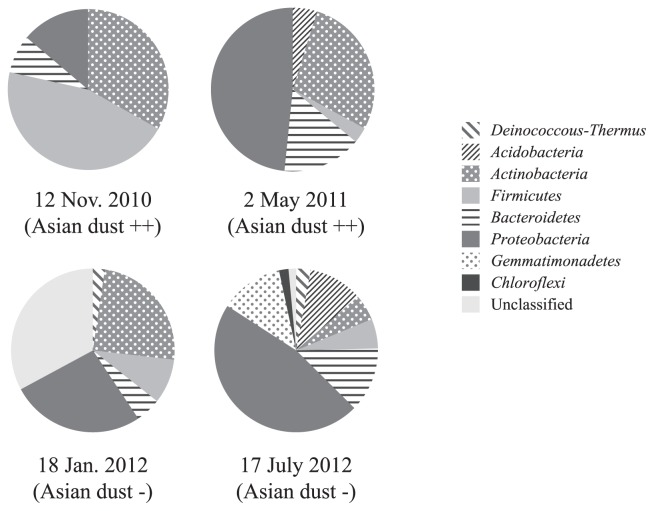
Relative abundance of the most common bacterial phyla of airborne bacteria in outdoor environments.

Airborne bacterial communities on Asian dust days and non-Asian dust days were further analyzed using a clone library to compare the phylogenetic compositions of bacteria in bioaerosols (Fig. 3). A rarefaction curve indicated that the expected richness at the phylum level was almost reached (Supplementary Fig. S3). Differences were observed in the airborne bacterial community compositions between two severe Asian dust days (12 November 2010 and 2 May 2011); however, *Actinobacteria* dominated both of these samples, accounting for 30% of the total bacterial community. *Firmicutes* dominated the bacterial community on 12 November 2010 (approximately 50% of the total), while *Proteobacteria* dominated on 2 May 2011 (approximately 50% of the total). These bacterial community compositions on Asian dust days also differed from those observed on non-Asian dust days (18 January 2012 and 17 July 2012). Marked differences were also noted in the airborne bacterial community compositions between these non-Asian dust days (Fig. 3).

The airborne bacterial community compositions of samples collected on these two Asian dust days differed from those in Asian dust collected over the Sea of Japan (10 km from the coast at an altitude of 900 meters), which had just reached the main island of Japan. The bacterial community composition of Asian dust collected on 12 November 2010 over the Sea of Japan at an altitude of 900 m by a small airplane was as follows (23): *Bacteroidetes* (28%), *Firmicutes* (23%), *Proteobacteria* (18%), *Actinobacteria* (16%), *Acidobacteria* (6%), *Deinococcus-Thermus* (2%), and others (7%), while that of bioaerosols collected in the outdoor environment (rooftop) was dominated by *Firmicutes* (45%). The bacterial community composition of Asian dust collected on 2 May 2011 over the Sea of Japan at 900 m altitude was as follows (23): *Firmicutes* (30%), *Bacteroidetes* (24%), *Actinobacteria* (23%), *Proteobacteria* (12%), *Acidobacteria* (5%), *Deinococcus-Thermus* (3%), and others (3%), while that of bioaerosols collected from an outdoor environment (rooftop) was dominated by *Proteobacteria* (48%). Severe Asian dust events delivered higher concentrations of bacteria (Table 1); therefore, the bacterial composition of Asian dust collected over the Sea of Japan and bioaerosols collected in outdoor environments should be similar if the bacteria in Asian dust events reached our sampling site. However, the results obtained in the present study indicate that this did not occur. Bioaerosols in outdoor environments were collected from the rooftop of a university building in the suburbs at a height of 20 m. The distance between the sampling point over the Sea of Japan and the university building is 160 km, and there are mountains (altitude: 700–1,200 m) as well as residential areas between them (Supplementary Fig. S4). When a severe Asian dust event occurred, the bacterial community in the Asian dust may have mixed with the indigenous airborne bacteria communities in these outdoor environments before reaching the sampling point on the rooftop. Culturable *Proteobacteria* found in Asian dust were similar to *Methylobacterium*, *Pseudomonas*, and *Enterobacteriales* (Fig. 2). These viable bacteria may be recent dust contaminants from the soil and wastewater treatment plants between the Sea of Japan and the sampling point (university building). Bacterial abundance in general soil environments is 10^9^–10^11^ cells g^−1^, and bacterial abundance in Asian dust was found to be 10^5^ cells m^−3^ (23). Therefore, mixing of even a small amount of soil particles with bioaerosols can change their community composition. Similar to our results, Smith *et al.* reported that Asian long-range transported air plumes delivered higher concentrations of microbes, while the bacterial community compositions in these air plumes were similar to those in the background air at their sampling point (Mt. Bachelor, Oregon, USA; 2,800 m above sea level) (19). Their sampling point was 13,000 km from the dust source region, and the bacterial community in this air plume was assumed to have markedly changed from the original community during this long-range transportation.

## Conclusions

In the present study, we demonstrated that bacteria in Asian dust did not immediately disturb airborne microbial communities in downwind areas 3,000–5,000 km from the dust source regions, even though large amounts of bacterial cells were transported by these severe atmospheric events. However, phylogenetically diverse bacterial groups with physiological activity are known to be transported by Asian dust events (23); therefore, it can be assumed that these transported bacteria affect the indigenous microbial ecosystems of large downwind areas such as Korea, Japan, and the Pacific islands on a geological time scale. Aeolian dust events may contribute to the global migration of bacterial cells and their genes, and can consequently be important sources of bacterial diversity in the earth’s ecosystem.

The results obtained in this study provide fundamental information for examining the effects of long-range transported bacteria on public health and ecosystems in downwind areas. The amount of Asian dust fallout is estimated to be 180 g m^−2^ year^−1^ in Beijing, China (17) and 1–10 g m^−2^ year^−1^ in Japan (24). Microbes in aeolian dust should have a greater influence on indigenous microbial community compositions in downwind areas near the dust source. Continuous temporal and spatial analyses from dust source regions to downwind regions (*e.g.*, from the Gobi desert to Beijing [China], Seoul [Korea], Osaka [Japan], Hawaii, and North America) will assist in estimating the impact of atmospherically transported bacteria on indigenous microbial ecosystems in downwind areas.

## Supplementary material



## Figures and Tables

**Table 1:** Abundance of airborne bacteria collected in outdoor environments determined by quantitative PCR targeting the 16S rRNA gene

Sample number	Sampling Date	Severity of Asian dust*	16S rRNA gene (copies/m^3^)	Estimated Bacterial number** (cells/m^3^)
A1	12 Nov. 2010	++	1×10^5^	6×10^3^–1×10^5^
A3	10 Apr. 2011	+	2×10^4^	1×10^3^–2×10^4^
A2	2 May 2011	++	2×10^5^	2×10^4^–2×10^5^
N1	15 Sep. 2011	−	1×10^3^	1×10^2^–1×10^3^
N2	7 Nov. 2011	−	5×10^2^	3×10^1^–5×10^2^
N3	15 Dec. 2011	−	4×10^3^	3×10^2^–4×10^3^
N4	18 Jan. 2012	−	3×10^3^	2×10^2^–3×10^3^
N5	7 Feb. 2012	−	2×10^3^	2×10^2^–2×10^3^
N6	6 Mar. 2012	−	3×10^3^	2×10^2^–3×10^3^
N7	12 Apr. 2012	−	7×10^3^	5×10^2^–7×10^3^
A4	24 Apr. 2012	+	4×10^3^	3×10^2^–4×10^3^
N8	26 Apr. 2012	−	1×10^4^	7×10^2^–1×10^4^
A5	16 May 2012	+	8×10^3^	1×10^2^–8×10^3^
N9	30 May 2012	−	7×10^2^	5×10^1^–7×10^2^
N10	17 Jul. 2012	−	1×10^3^	8×10^1^–1×10^3^

* Determined by information obtained from the Japan Meteorological Agency, LIDAR, and visibility at the sampling site. ++: severe dust event, +: moderate dust event, −: no dust event.

** Bacterial cells carry 1–15 copies of the 16S rRNA gene in their genome.

## References

[1] Bishop JKB, Davis RE, Sherman JT (2002). Robotic observations of dust storm enhancement of carbon biomass in the North pacific. Science.

[2] Bory AJ-M, Biscaye PE, Grousset FE (2003). Two distinct seasonal Asian source regions for mineral dust deposited in Greenland (NorthGRIP). Geophys Res Lett.

[3] Cole JR, Wang Q, Cardenas E (2009). The Ribosomal Database Project: improved alignments and new tools for rRNA analysis. Nucleic Acids Res.

[4] Duce RA, Unni CK, Ray BJ, Prospero JM, Merrill JT (1980). Long-range atmospheric transport of soil dust from Asia to the tropical North Pacific: temporal variability. Science.

[5] Edwards U, Rogall T, Blöcker H, Emde M, Böttger EC (1989). Isolation and direct complete nucleotide determination of entire genes. Characterization of a gene coding for 16S ribosomal RNA. Nucleic Acids Res.

[6] Griffin DW (2007). Atmospheric movement of microorganisms in clouds of desert dust and implications for human health. Clin Microbiol Rev.

[7] Griffin DW, Garrison VH, Herman JR, Shinn EA (2001). African desert dust in the Caribbean atmosphere: microbiology and public health. Aerobiologia.

[8] Griffin DW, Kellogg CA, Garrison VH, Lisle JT, Borden TC, Shinn EA (2003). Atmospheric microbiology in the northern Caribbean during African dust events. Aerobiologia.

[9] Grousset FE, Ginoux P, Bory A, Biscaye PE (2003). Case study of a Chinese dust plume reaching the French Alps. Geophys Res Lett.

[10] Hervàs A, Camarero L, Reche I, Casamayor EO (2009). Viability and potential for immigration of airborne bacteria from Africa that reach high mountain lakes in Europe. Environ Microbiol.

[11] Iwamoto T, Tani K, Nakamura K, Suzuki Y, Kitagawa M, Eguchi M, Nasu M (2000). Monitoring impact of in situ biostimulation treatment on groundwater bacterial community by DGGE. FEMS Microbiol Ecol.

[12] Iwasaka Y, Iwasaka Y (2006). Pathway of Asian dust. Asian Dust.

[13] Iwasaka Y, Minoura H, Nagaya K (1983). The transport and spacial scale of Asian dust-storm clouds: a case study of the dust-storm event of April 1979. Tellus.

[14] Iwasaka Y, Yamato M, Imasu R, Ono A (1988). The transport of Asian dust (KOSA) particles; importance of weak KOSA events on the geochemical cycle of soil particles. Tellus.

[15] Kawai M, Matsutera E, Kanda H, Yamaguchi N, Tani K, Nasu M (2002). 16S ribosomal DNA-based analysis of bacterial diversity in purified water used in pharmaceutical manufacturing processes by PCR and denaturing gradient gel electrophoresis. Appl Environ Microbiol.

[16] Kellogg CA, Griffin DW (2006). Aerobiology and the global transport of desert dust. Trends Ecol Evol.

[17] Nishikawa M, Mori I, Di Y, Quan H (2002). Source impacts of fall-out dust in Beijing. Proc Internat Aerosol Conference Taiwan.

[18] Prospero JM, Blades E, Mathison G, Naidu R (2005). Interhemispheric transport of viable fungi and bacteria from Africa to the Caribbean with soil dust. Aerobiologia.

[19] Smith DJ, Timonen HJ, Jaffe DA, Griffin DW, Birmele MN, Perry KD, Ward PD, Roberts MS (2012). Intercontinental dispersal of bacteria and archaea in transpacific winds. Appl Environ Microbiol.

[20] Tsai YL, Olson BH (1991). Rapid method for direct extraction of DNA from soil and sediments. Appl Environ Microbiol.

[21] Uno I, Eguchi K, Yumimoto K (2009). Asian dust transported one full circuit around the globe. Nature Geosci.

[22] Wilson KH, Blitchington RB, Greene RC (1990). Amplification of bacterial 16S ribosomal DNA with polymerase chain reaction. J Clin Microbiol.

[23] Yamaguchi N, Ichijo T, Sakotani A, Baba T, Nasu M (2012). Global dispersion of bacterial cells on Asian dust. Sci Rep.

[24] Yoshinaga S (1998). Accumulation rate of tropospheric dust in and around the Japan Islands during the latest quaternary. Quaternary Research.

