# Supplementary material for: Changes in the Airborne Bacterial Community in Outdoor Environments following Asian Dust Events
Source: Microbes Environ. 2014 Feb 19;29(1):82–8. doi: 10.1264/jsme2.ME13080 (PMC4041233; doi:10.1264/jsme2.ME13080)
Supplement: Supplementary file 1 [file 29_82_s1.pdf]

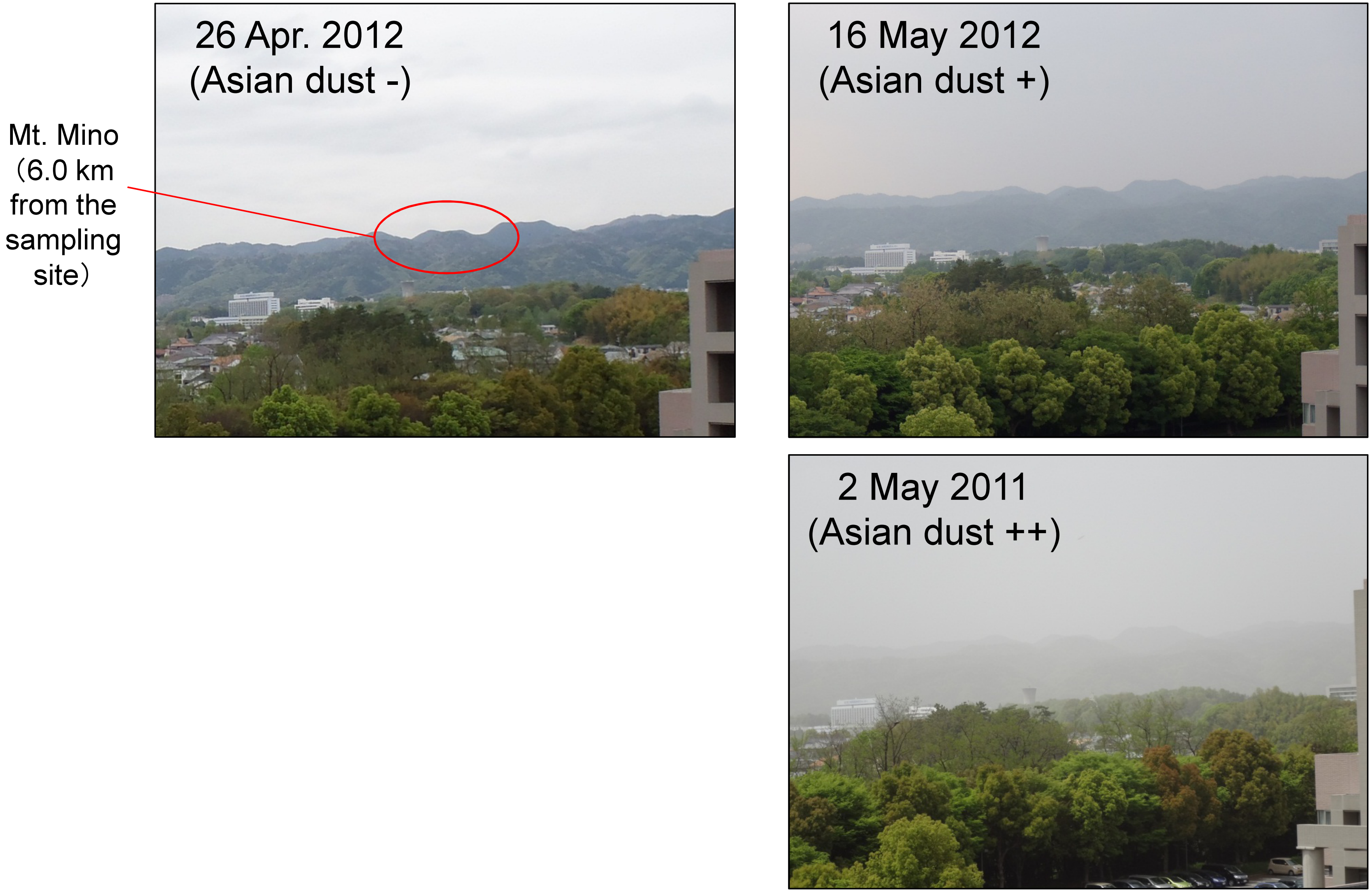

Supplementary Figure S1. View from the sampling point.

12 November 2010

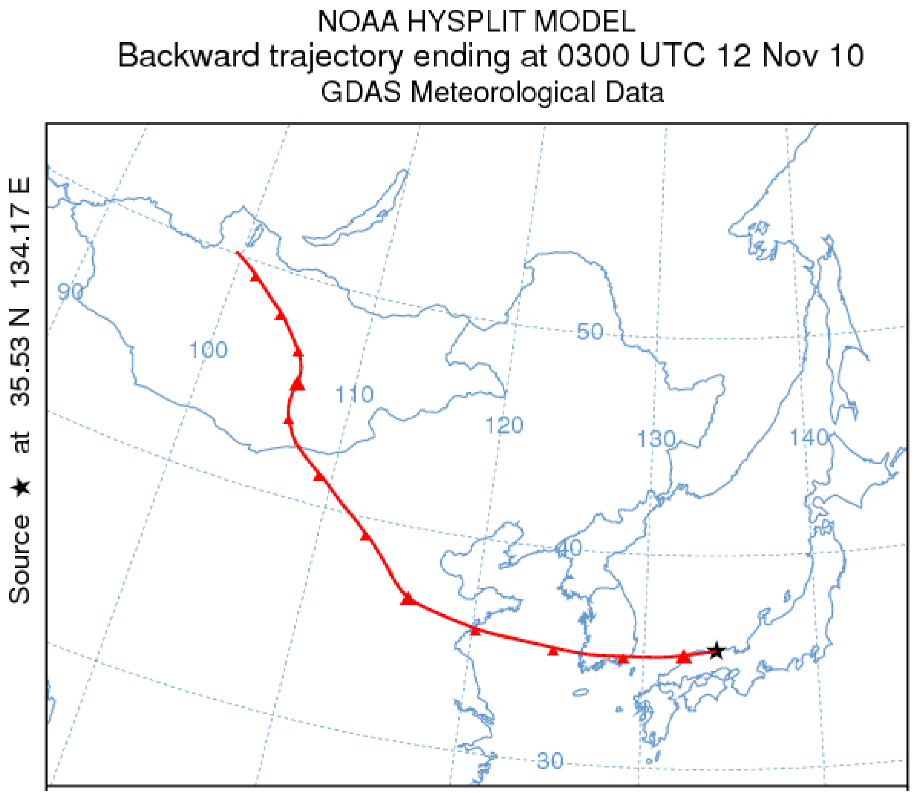

2 May 2011

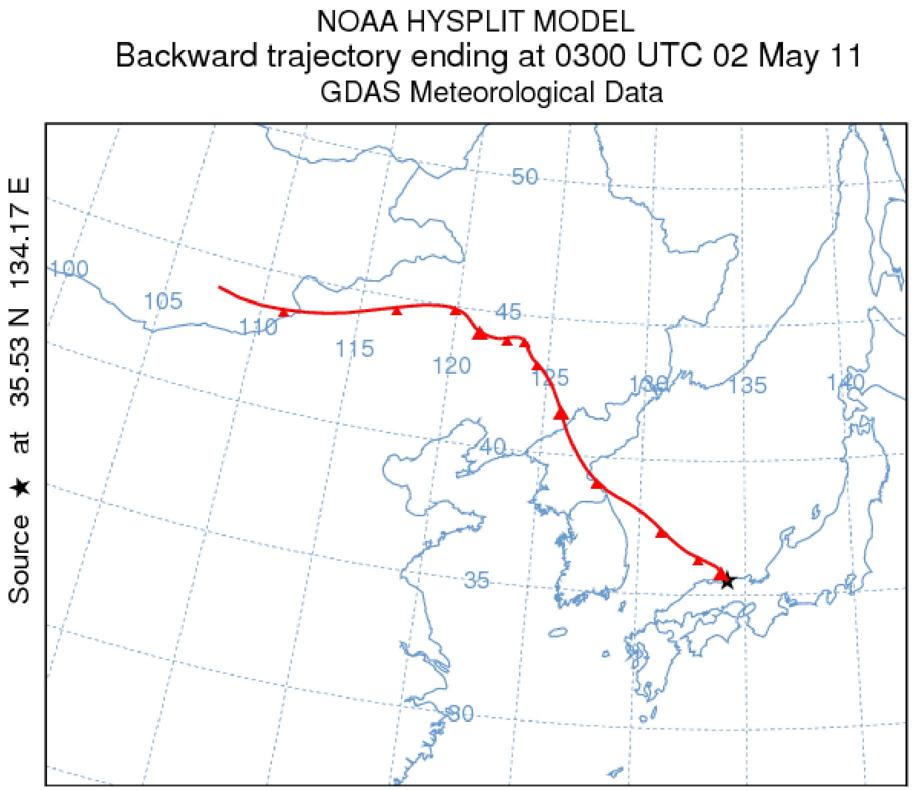

Parameters:

End time: 0300 UTC on each sampling day
Latitude: 35.53 N, Longitude: 134.17 E
Height: 900 m, Duration: 72 hours

(http://ready.arl.noaa.gov/HYSPLIT.php)

Supplementary Figure S2. Source region of Asian dust storm commencing 12 Nov. 2010 and 2 May 2011 in Japan, estimated by backward trajectory analysis.

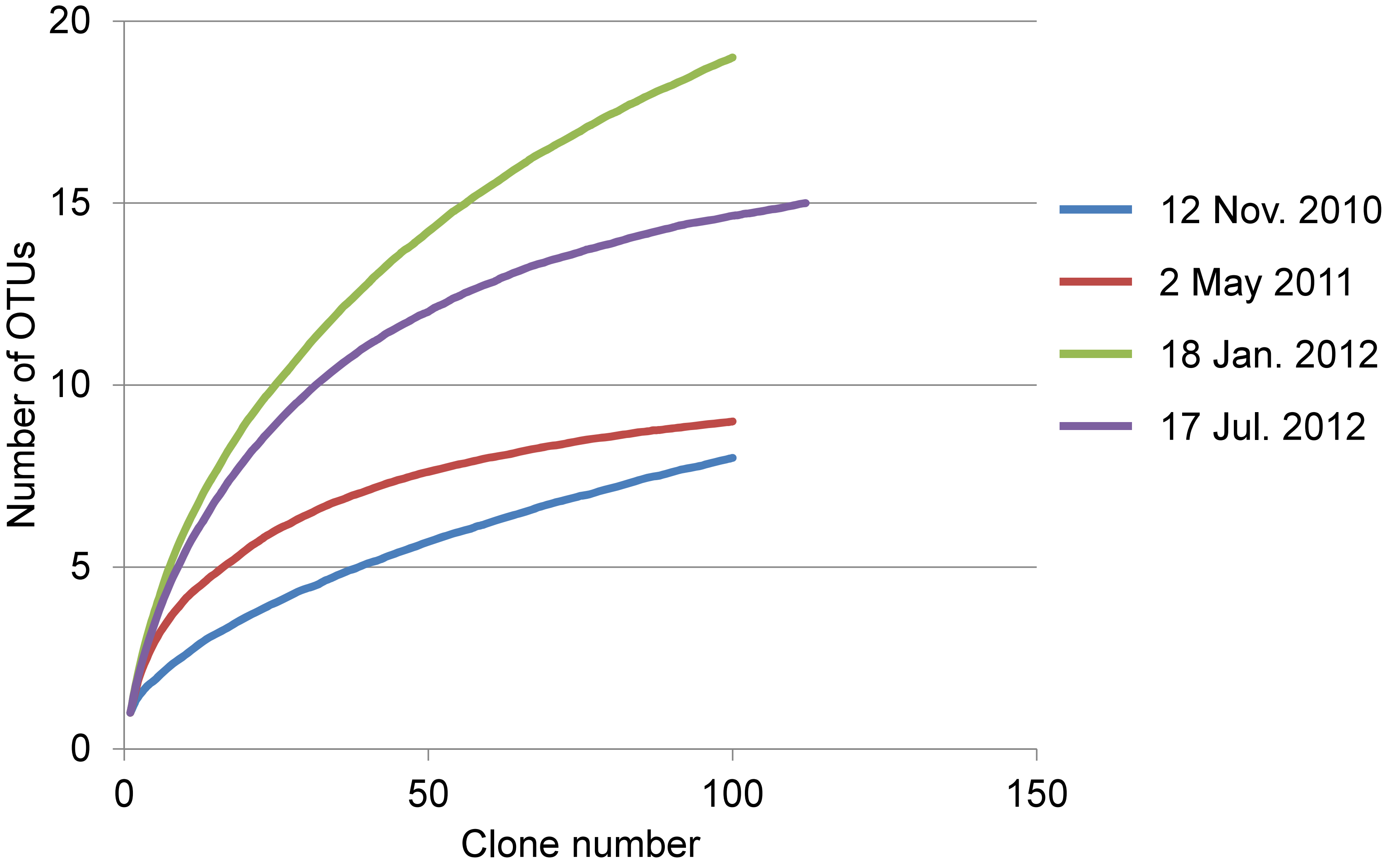

Supplementary Figure S3. Rarefaction curves of phylum richness of airborne bacteria in outdoor environment.

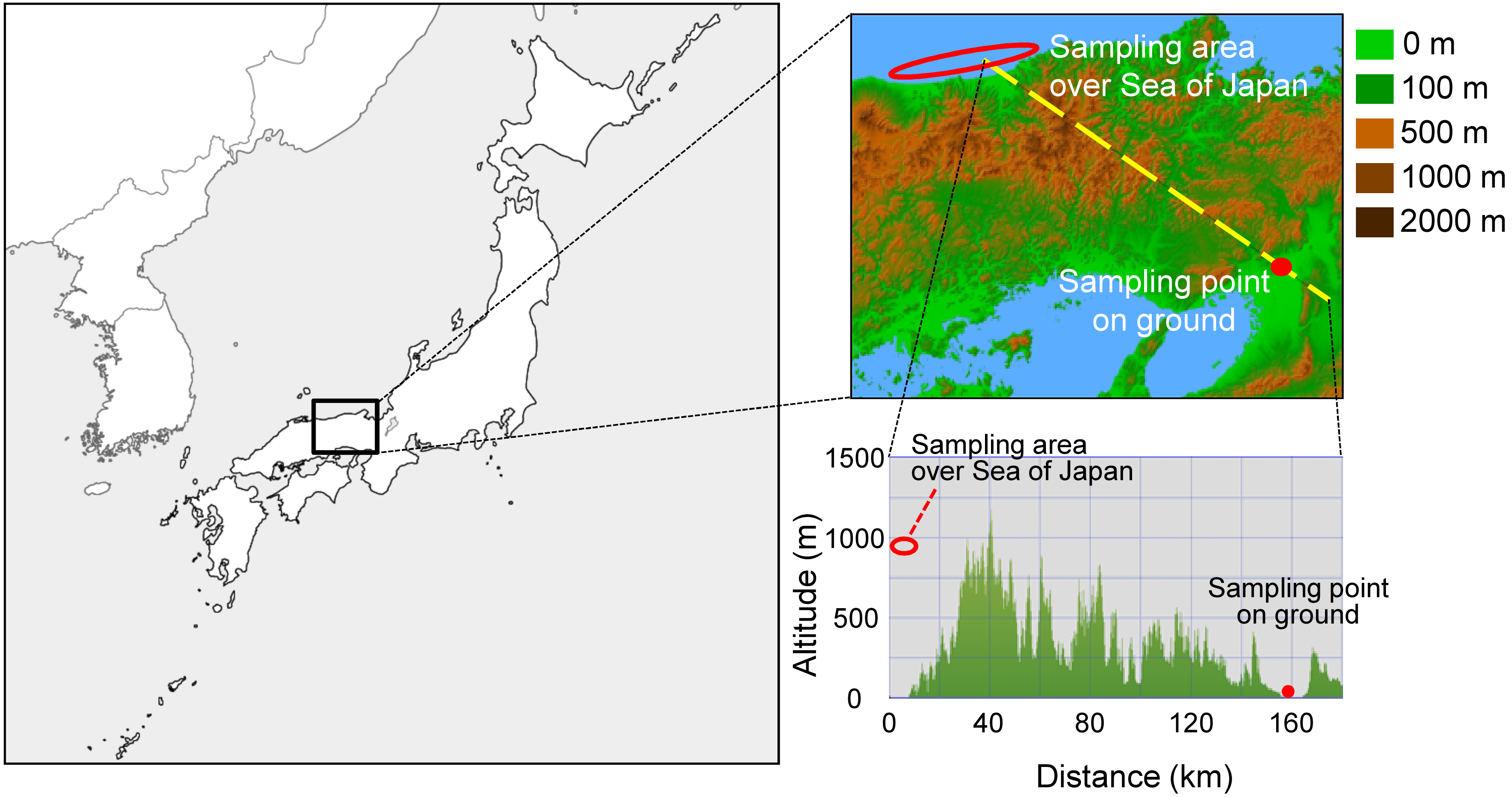

Supplementary Figure S4. Map and its profile of the two sampling points.
